# Beta_2_-agonist and anti-inflammatory combination therapy for asthma rescue: a paradigm shift in asthma care

**DOI:** 10.3389/fmed.2026.1826737

**Published:** 2026-06-01

**Authors:** Neil Skolnik, Jessica Stieritz, Julie Akers, Timothy Hudd, Wendy L. Wright, Ileen Gilbert, Nazrin Yusufova, Bill Cook, Emily Robb, Henry Naddaf

**Affiliations:** 1 Sidney Kimmel Medical College, Jenkintown, PA, United States; 2 College of Pharmacy and Pharmaceutical Sciences, Washington State University, Spokane, WA, United States; 3 Massachusetts College of Pharmacy and Health Sciences, Boston, MA, United States; 4 Wright & Associates Family Healthcare, Amherst, NH, United States; 5 BioPharmaceuticals, United States Medical, AstraZeneca, Wilmington, DE, United States; 6 BioPharmaceuticals, Global Medical Affairs, AstraZeneca, Gaithersburg, MD, United States; 7 Ernest Mario School of Pharmacy, Rutgers University, Newark, NJ, United States; 8 The Toledo Clinic, Family Medicine, Toledo, OH, United States

**Keywords:** asthma, exacerbation, inhaled corticosteroids, rescue therapy, short-acting beta_2_-agonists

## Abstract

The Global Initiative for Asthma (GINA) report and national and international guidelines have shifted away from short-acting beta_2_-agonist (SABA)-only bronchodilators as first-line asthma rescue therapy, recognizing that both bronchoconstriction and inflammation underlie the increase in symptoms preceding an exacerbation. Instead, the GINA Committee supports strategies to reduce inflammation, such as anti-inflammatory reliever (AIR) therapy (as-needed, low-dose inhaled corticosteroid [ICS]–formoterol or SABA–ICS for symptom relief) and single maintenance and reliever therapy (SMART) or maintenance and reliever therapy (MART; i.e., ICS–formoterol used as both maintenance and as-needed rescue therapy). This narrative review discusses the rationale behind the move toward AIR and SMART regimens, examining the inflammatory pathophysiology of exacerbations and how the ramp-up phase may represent a “window of opportunity” for prompt anti-inflammatory treatment to prevent exacerbations. Key clinical data from studies of AIR and SMART regimens, as well as the importance of as-needed SABA–ICS use in routine clinical practice, are also discussed. The review highlights the importance of ICS delivery during times of wheezing and/or symptoms to reduce the ongoing inflammatory cascade, thereby reducing the likelihood of initial symptoms developing into an exacerbation. In this way, patients can avoid the need for systemic corticosteroids, reducing the overall lifetime steroid load and the risk of lifetime dose-dependent complications such as bone thinning, diabetes, hypertension, and many other conditions.

## 1 Introduction

Asthma is the most common chronic respiratory disease in the United States (US), affecting 8.2% of the total population, including approximately 22 million adults (aged ≥18 years) and 4.5 million children (1). In addition, asthma is uncontrolled, defined as poor symptom control and/or frequent exacerbations, in approximately 62% of adults and 50% of children (2). Primary care physicians (PCPs) play an integral role in managing asthma, given that most (≥60%) patients with asthma, including those with uncontrolled asthma, have their disease managed in primary care settings (3, 4).

Asthma is characterized by repeated episodes of wheezing, breathlessness, chest tightness, and coughing and represents a significant health and economic burden (5, 6). During asthma exacerbations, patients experience a decline in lung function leading to sudden or progressive worsening of symptoms (4). The primary goals of asthma management include reducing symptom burden and minimizing the risk of exacerbations (7).

Traditionally, short-acting beta_2_-agonists (SABAs), most commonly albuterol in the US (7), have been used as the first step and primary intervention for rescue management when symptoms increase, despite recognition that asthma is an inflammatory disease (8). This creates a paradox: patients rely on bronchodilator-only treatment for symptoms, which relieves bronchoconstriction but allows the increasing inflammation to continue unabated (9). The delivery of an anti-inflammatory rescue medication directly to the airway during times of bronchoconstriction provides the opportunity to decrease ongoing inflammation in the airway and blunt the progression of the symptoms from escalating to a severe exacerbation, while maintaining bronchodilation with albuterol (10).

While SABA monotherapy continues to be widely used due to perceived efficacy by patients, the Global Initiative for Asthma (GINA) has discouraged patients from relying solely on a SABA since 2019 due to its association with an increased risk of severe exacerbations and mortality (11). Moreover, as adherence to inhaled corticosteroid (ICS) maintenance therapy remains suboptimal (12), GINA now supports treatment strategies that target airway inflammation by incorporating ICS into rescue therapy across all asthma severities (7). For patients aged ≥12 years with mild asthma, anti-inflammatory reliever (AIR) therapy options include as-needed combination therapy with ICS–formoterol or SABA–ICS (7). In patients with moderate-to-severe asthma, single maintenance and reliever therapy (SMART; or maintenance and reliever therapy [MART]) with ICS–formoterol allows a single inhaler to serve as both the maintenance and as-needed medication in asthma (7). Descriptions of the various treatment terminologies and regimens are provided in Table 1 (7).

**Table 1:** Terminology for asthma medications (7).

Term/acronym	Definition	When applicable	Key notes/limitations
SABA	Traditional rescue inhaler (e.g., albuterol); provides rapid bronchodilation without anti-inflammatory effect	Historically used as rescue therapy across all asthma severities	No longer recommended to be used as monotherapy in asthma treatment Should be paired with an ICS when treating and preventing symptoms
ICS	Anti-inflammatory maintenance inhaler	Used across all GINA steps of asthma care	Low-dose ICS combined with fast-acting bronchodilators are preferred for treating symptoms and preventing exacerbations
SMART (or MART)	Single inhaler containing ICS and formoterol used for both maintenance and rescue therapy	Used for GINA steps 3–5 in adults and adolescents	ICS–formoterol is not FDA-approved for rescue or rescue and maintenance use in the US As a rescue treatment, not maintenance ICS–LABA agnostic Cannot be used with currently approved triple therapy (ICS–LABA–LAMA)
AIR	As-needed rescue inhaler combining a rapid-onset bronchodilator with an anti-inflammatory (e.g., SABA–ICS or off-label ICS–formoterol)	SABA–ICS used for GINA steps 1–5 in patients aged ≥18 years	SABA–ICS can be used alongside any maintenance regimen (i.e., maintenance agnostic)
Triple therapy (ICS–LABA–LAMA)	Inhaler combining three drug classes: corticosteroid, LABA, and LAMA	Used in GINA step 5 for severe asthma, when asthma remains uncontrolled on high-dose SABA–ICS	Can be used alongside AIR with SABA–ICS Cannot be used as SMART or with ICS–formoterol as rescue

AIR, anti-inflammatory reliever; FDA, US Food and Drug Administration; ICS, inhaled corticosteroid; LABA, long-acting beta_2_-agonist; LAMA, long-acting muscarinic antagonist; MART, maintenance and reliever therapy; SABA, short-acting beta_2_-agonist; SMART, single maintenance and reliever therapy.

ICS–formoterol is approved to be used as part of a SMART (MART) regimen in over 120 countries (13) and as rescue therapy alone in 48 countries, including the United Kingdom (14). ICS–formoterol is only available in the US as a pressurized metered-dose inhaler (pMDI), and it is not approved for either SMART (MART) use or as rescue therapy. However, a SABA–ICS fixed-dose combination inhaler containing albuterol–budesonide is approved in the US and currently represents the only ICS-based rescue therapy option (15). Unlike ICS–formoterol, which is available in multiple formulations, dosages, and devices, depending on the country and regulatory approval, albuterol–budesonide is only available as a pMDI (15) Clinical trials have demonstrated that albuterol–budesonide reduces the risk of severe exacerbations across asthma severity (16, 17).

The objective of this narrative review is to discuss the rationale and clinical data supporting the change in the rescue therapy paradigm that now includes an ICS plus a fast-acting bronchodilator for patients with asthma across the spectrum of disease severity, with a focus on adult patients.

## 2 Epidemiology of exacerbations

**Figure 1:**
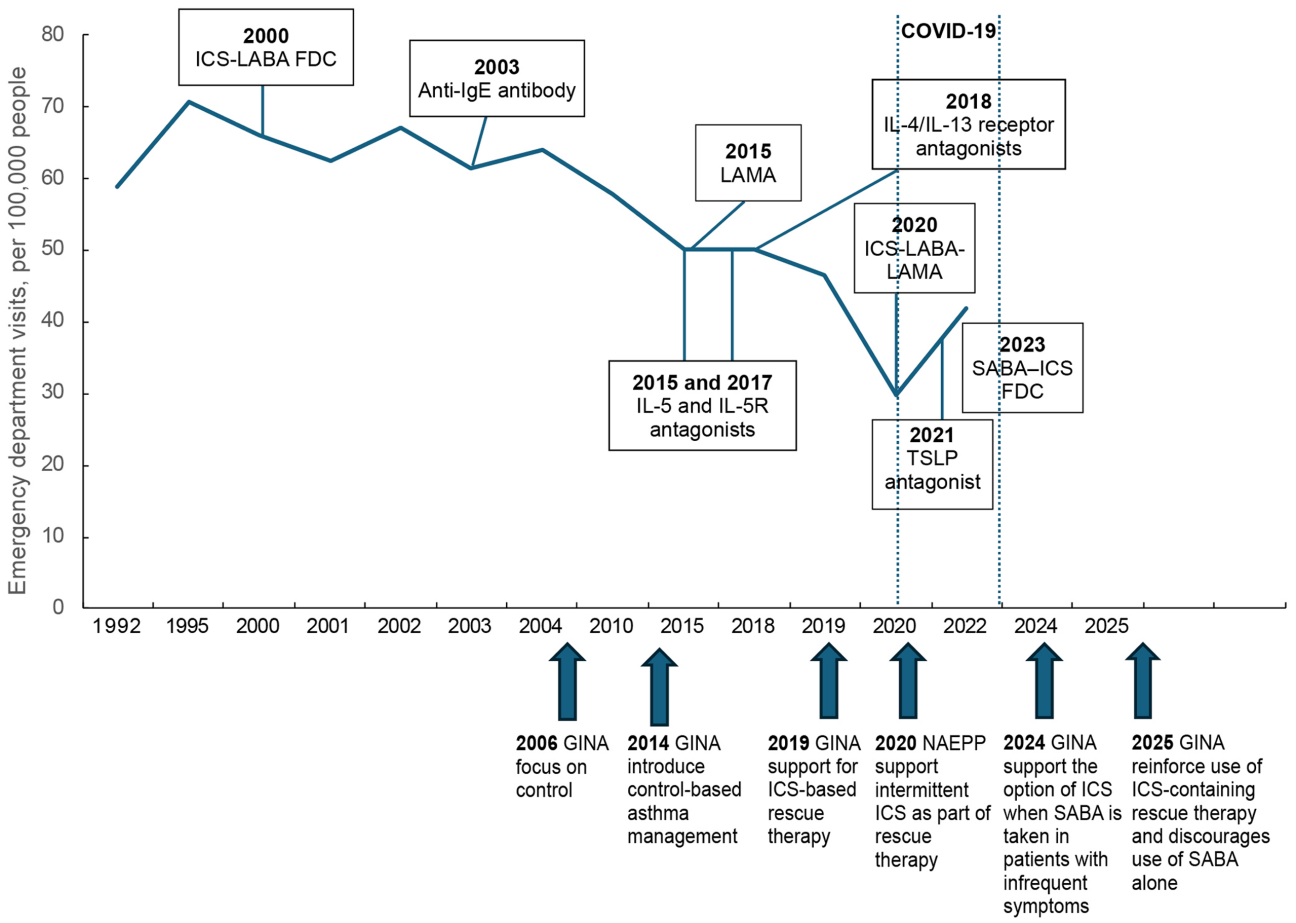
Trajectory of asthma-related ED visits and the effects of key asthma treatment milestones and other events (11, 15, 18–30). ED, emergency department; FDC, fixed-dose combination; GINA, Global Initiative for Asthma; ICS, inhaled corticosteroid; Ig, immunoglobulin; IL, interleukin; IL-5R, interleukin 5 receptor; LABA, long-acting beta_2_-agonist; LAMA, long-acting muscarinic antagonist; NAEPP, National Asthma Education and Prevention Program; SABA, short-acting beta_2_-agonist; TSLP, thymic stromal lymphopoietin.

The rate of asthma exacerbations increased in the 1980s, peaked in the 1990s, and steadily declined over the 2000s and 2010s (Figure 1) (11, 15, 18–30). However, the prevalence of exacerbations only declined by approximately 10 percentage points between 2010 and 2020 (2). The decrease in exacerbation rate over time has been attributed to use of emerging evidence-based strategies in asthma diagnosis, management, and treatment and ongoing public health programs for clinicians and patients (2, 31). The coronavirus disease (COVID-19) pandemic further suppressed exacerbations, possibly due, in part, to reduced exposure to respiratory pathogens and asthma triggers because of stay-at-home orders and social distancing efforts (2, 32). However, more recent reports suggest emergency department (ED) visits due to asthma exacerbations have increased post-pandemic (2, 32).

Multiple factors are known to play a role in asthma exacerbations. Among these, lower socioeconomic status and other social determinants have consistently been associated with higher rates of exacerbation, ED visits, and hospitalizations (33–35). Potential contributing factors include higher smoking rates, limited access to health care and medications, poorer housing conditions, and less effective self-management (33–35). In addition, poor adherence to ICS maintenance therapy (12), underutilization of asthma action plans, and incorrect inhaler technique have also been associated with an increased risk of exacerbations (12, 36, 37), ED visits (12), and poor asthma control (36). Together, these factors may explain why real-world exacerbation rates remain higher than those reported in clinical trials, which typically have high treatment adherence, structured monitoring, and access to medical therapies (38).

Suboptimal adherence to ICS-based maintenance therapy remains a persistent challenge in asthma care, with 24% of exacerbations and 60% of hospitalizations attributed to poor adherence (39). Multiple factors contribute to poor adherence to ICS-based maintenance therapy, including insufficient understanding of its role, concern about its safety, and a tendency to deprioritize it because it does not provide quick relief (40). Therefore, it is important to explain to patients what asthma is and how inhaled medications work, using airway diagrams and models as teaching aids. PCPs should also reiterate to their patients that frequent use of an as-needed SABA is an indication of poor asthma control, and that SABA-only therapy does not address the underlying inflammation, making them more susceptible to exacerbations (40).

In addition to adherence, inhaler technique should also be routinely assessed, as correct delivery of medication to the airways is essential for asthma control (40). Common inhaler errors include improper device preparation, incorrect inhalation type for the prescribed device, and failure to hold the breath after inhalation. Accordingly, PCPs should ask patients to demonstrate their inhaler technique during office visits and, if errors are identified, model correct use using a placebo device (7, 40).

Despite the development of newer maintenance therapies based upon a better understanding of the pathophysiology of asthma, and regardless of the inciting factors, asthma exacerbations continue to impact morbidity and mortality. Asthma exacerbations remain a major cause of ED visits and have a negative impact on health-related quality of life (41). In 2019, there were 4.9 million physician office visits for asthma in the US (42). In 2020, asthma accounted for 986,453 ED visits and 94,560 hospitalizations in the US (43); in 2021, there were 3,517 asthma-related deaths (1). Projected 20-year (2019–2038) total direct and indirect costs associated with uncontrolled asthma in patients aged ≥15 years have been estimated at US$963.5 billion (44).

Acute use of a systemic corticosteroid (SCS) for exacerbations remains common, with a minority of patients relying on a chronic SCS to control symptoms. While a SCS is necessary and effective for treating the underlying increase in airway inflammation that occurs during exacerbations and for the short-term resolution of the associated symptoms (45–47), the cumulative levels of SCS exposure that can occur with repetitive exacerbations have been linked to adverse health outcomes that increase with increasing dose and duration of SCS treatment (45–48). Short-term or intermittent use, even at moderate doses (e.g., ≥10–20 mg prednisone-equivalent daily), may cause acute adverse effects within the first 30 days of use, including sepsis, venous thromboembolism, and fracture (49). With repeated courses or long-term use (i.e., >3 months or cumulative exposure starting at 500 to <1,000 mg prednisone-equivalent), the risk for other adverse effects increases, including infection, osteoporosis, adrenal suppression, diabetes mellitus, sleep disturbance, cataracts, obesity, and cardiovascular disease (46, 48, 49).

It is important to note that exacerbations can occur regardless of asthma severity, with one real-world study reporting ≥1 severe exacerbation per year in 61.2% of 373,471 patients aged ≥12 years with intermittent asthma who were receiving SABA-only therapy (50, 51). Reducing the risk of exacerbations has the potential to greatly improve morbidity, mortality, and health-related quality of life, even in patients with mild asthma.

## 3 Clinical features of asthma and asthma exacerbations

Foundational therapy for asthma management has long included daily use of an ICS to decrease the airway inflammation that is characteristic of asthma (7). Addition of a long-acting beta_2_-agonist (LABA) to an ICS also reduces the risk of exacerbations and improves lung function (52). For decades, rescue therapy with a SABA alone was thought to be sufficient to address bronchoconstriction. However, more recently, the combination of an ICS with a fast-acting bronchodilator has been found to be a better option to concomitantly treat the inflammation and bronchoconstriction that underlie the symptoms that can lead to an exacerbation (53).

**Figure 2:**
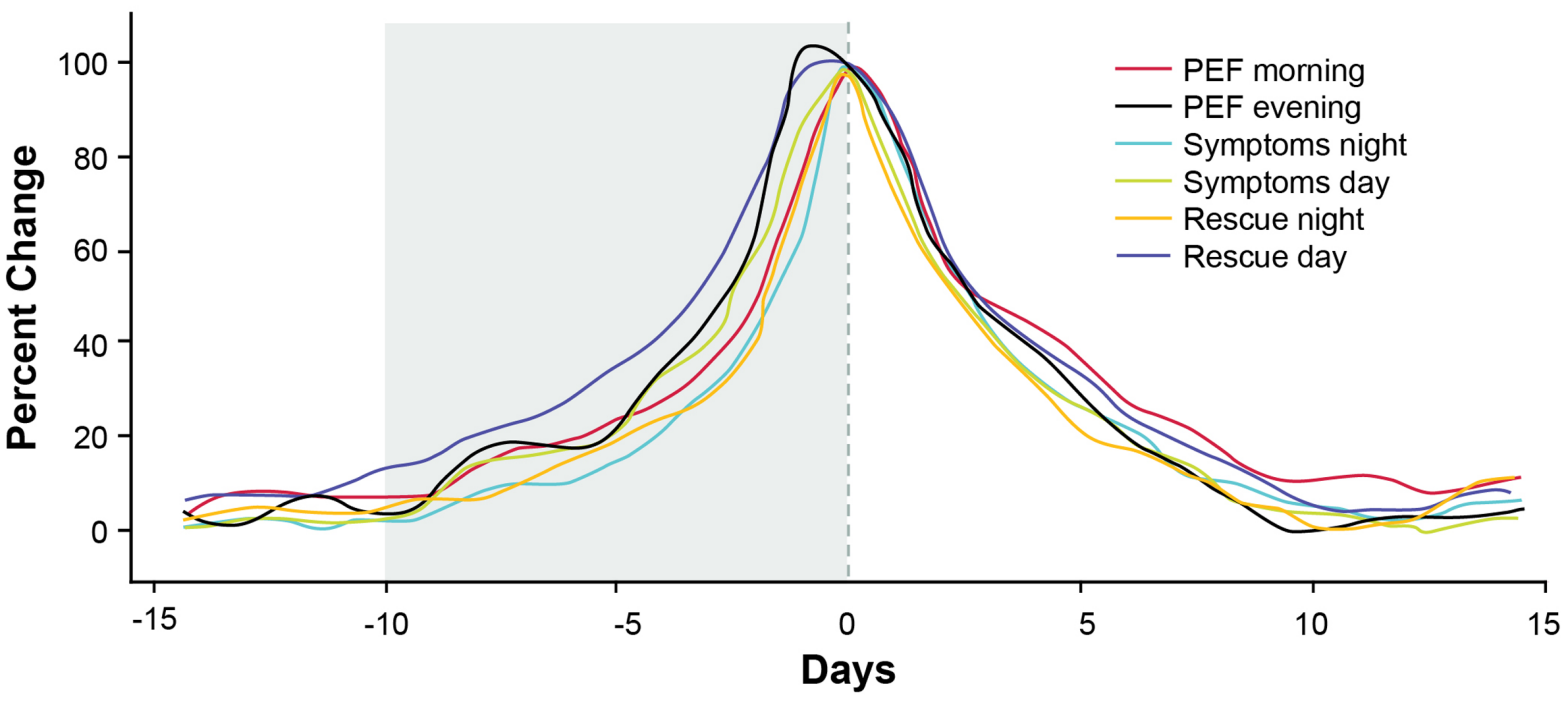
Ramp-up to an asthma exacerbation, showing changes in morning and evening PEF, day- and night-time symptoms, and rescue beta_2_-agonist use during an exacerbation (52). Patients aged ≥18 years using ICS or ICS + LABA maintenance therapy. To compare the rate of change in PEF, symptoms, and bronchodilator use before an exacerbation, the data were standardized by expressing the measurement on Day-14 as 0% and on Day 0 as 100%. ICS, inhaled corticosteroid; LABA, long-acting beta_2_-agonist; PEF, peak expiratory flow. From (52), © 1999 The American Thoracic Society; Adapted with permission of Oxford University Press.

As shown in a follow-up analysis of the FACET study that explored the impact of adding a LABA to an ICS as maintenance therapy for symptomatic patients, asthma exacerbations are often preceded by a 14-day ramp-up phase in which there is a 5–7-day gradual decline in peak expiratory flow (PEF) followed by a 2–3-day rapid decline (Figure 2) (52). In this study, the PEF decline was mirrored by worsening symptoms and increased use of rescue SABAs (52).

**Figure 3:**
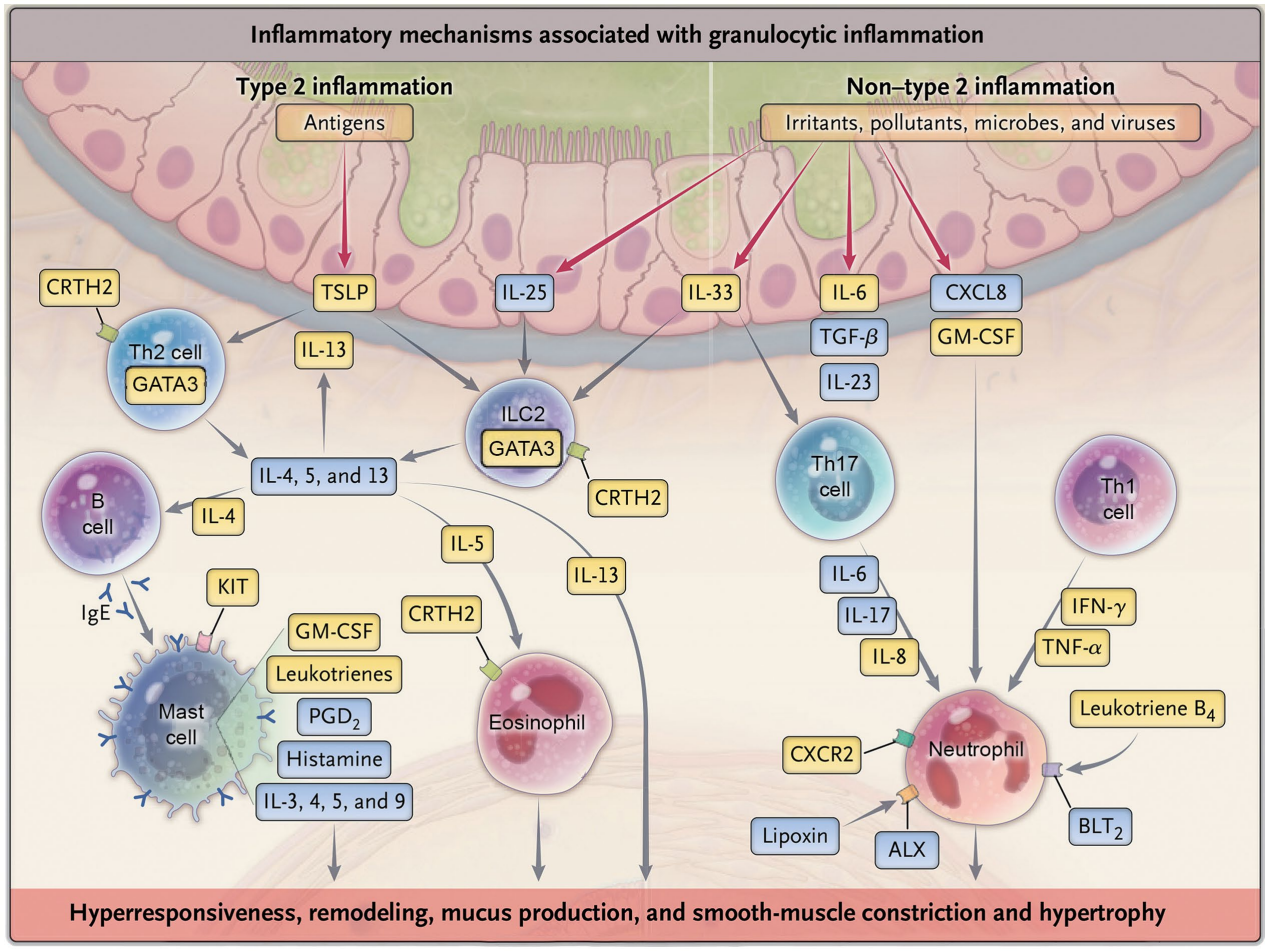
The inflammatory mechanisms that contribute to the pathophysiology of severe asthma are heterogeneous (56). ALX, lipoxin A4 receptor; BLT2, leukotriene B4 receptor 2; CRTH2, chemoattractant receptor-homologous molecule expressed on Th2; CXCL8, CXC ligand 8; CXCR2, CXC chemokine receptor 2; GATA3, GATA binding protein 3; GM-CSF, granulocyte-macrophage colony-stimulating factor; IFN, interferon; IL, interleukin; ILC2, group 2 innate lymphoid cells; Ig, immunoglobulin; KIT, tyrosine kinase receptor; PGD2, prostaglandin D2; TGF-β, transforming growth factor β; Th, T helper; TNF-α, tumor necrosis factor α; TSLP, thymic stromal lymphopoietin. From (56); Copyright © (2017) Massachusetts Medical Society. Reprinted with permission from Massachusetts Medical Society.

During this ramp-up period, airway inflammation and hyper-responsiveness can be amplified by a cascade of events, starting with epithelial cells releasing several alarmins, including interleukin (IL)-33, IL-25, and thymic stromal lymphopoietin. These alarmins most often initiate T2 inflammation by recruiting and activating group 2 innate lymphoid cells (ILC2) and amplifying T helper 2 (Th2) responses. Th2 and ILC2 release cytokines IL-4, IL-5, and IL-13 (54, 55) (Figure 3) (56), ultimately driving the hallmark symptoms of asthma, including wheezing, shortness of breath, variable airflow limitation, and airway hyperresponsiveness (54).

## 4 Window of opportunity

The ramp-up phase of inflammation represents a “window of opportunity” for treatment with an anti-inflammatory rescue medication to help address increasing inflammation to reduce the risk of asthma exacerbations (4, 51). SABA therapy had been recommended as the preferred rescue therapy in the past (20, 57), since bronchoconstriction is the primary driver of asthma symptoms (58). However, SABA-only rescue therapy leaves the underlying airway inflammation untreated (59).

Consequently, unaddressed inflammation may increase the risk of exacerbations, even in patients with well-controlled asthma and low SABA use (i.e., a single SABA cannister fill per year) (60). Furthermore, abundant evidence suggests that overuse of SABA-only rescue therapy—defined as ≥3 prescribed 200-dose canisters per year (i.e., an average of ≥1.5 puffs per day) (7)—is associated with an increased risk for various adverse outcomes, including severe exacerbations, hospitalizations, and death (Table 2) (7, 51, 57, 59–67). Therefore, it is important to consider SABA use relative to ICS administration (63); SABA use without regular ICS administration temporarily improves symptoms without targeting inflammation (68). Despite the potential adverse outcomes, reliance on SABA therapy and underuse of ICS maintenance therapy persist (68).

**Table 2:** Adverse outcomes associated with SABA therapy across asthma severities (51, 60–62, 65–67).

Study name, author year	Outcome measure	Asthma severity	Number of SABA canisters/year	Outcome
SABINA Europe and North America, Quint 2022 (67)	Severe exacerbations (US commercially insured population)	GINA Steps 1–2	1–2 (versus ≥3)	IRR (95% CI) 0.92 (0.91, 0.93)
SABINA Europe, Nwaru 2020 (66)	Severe exacerbation Overall mortality	GINA Steps 1–4	3–5 (versus ≤2)	HR (95% CI) 1.26 (1.24, 1.28) HR (95% CI) 1.26 (1.14, 1.39)
SABINA Canada, Noorduyn 2022 (65)	Severe exacerbation	GINA Steps 1–5	≥3 (versus <3)	IRR (95% CI) 1.36 (1.18, 1.56)
SABINA Hong Kong, Fung 2024 (62)	All-cause mortality Hospital admission	Across all severities	3–6 (versus ≤2)	aHR (95% CI) 1.22 (1.00, 1.50) aRR (95% CI) 2.74 (2.16, 3.49)
SABINA III Global, Bateman 2022 (61)	Severe exacerbation	GINA Steps 1–5	3–5 (vs 1–2)	aIRR (95% CI) 1.40 (1.24, 1.58)
US real-world patterns, Lugogo 2021 (60)	Exacerbations	GINA Steps 2–5	1	Mean (SD) annual exacerbation rate/person/year; percent of patients with ≥1 exacerbation 1.0 (1.45); 45.8
US retrospective cohort study, Lugogo 2025 (51)	Exacerbation	GINA Step 1	1 (vs 2–3 or ≥4)	Proportion of patients with ≥1 exacerbations was highest for those receiving 1 SABA fill (70.4%) vs. 2–3 SABA fills (52.5%) or ≥4 SABA fills (52.8%) (*p* < 0.001)

aHR, adjusted hazard ratio; aIRR, adjusted incidence rate ratio; aRR, adjusted rate ratio; HR, hazard ratio; IRR, incidence rate ratio; SABA, short-acting beta_2_-agonist; SD, standard deviation.

Incorporating an ICS into rescue therapy may help to address inflammation during the window of opportunity (7, 68). Historically, the anti-inflammatory effects of ICS were attributed mainly to genomic mechanisms, which take hours to days to take effect (Table 3) (69–74). However, it is well established that ICS also exert rapid nongenomic effects, which occur within minutes via interactions with the cell membrane, cytosolic glucocorticoids, or membrane-bound glucocorticoids, resulting in bronchorelaxation (69, 71–74). The immediate bronchorelaxation and delayed anti-inflammatory actions of ICS therapy underpin its role not only in long-term control but also as a first-line response strategy during symptom worsening, interrupting the inflammatory cascade before it escalates and potentially altering the course of an impending exacerbation (69, 73).

**Table 3:** Genomic and nongenomic effects of inhaled corticosteroids (69–74).

Factor	Genomic effects	Nongenomic effects
Onset	Hours to days	Minutes
Mechanism	Binding to cytoplasmic GRs → nuclear translocation → gene transcription modulation	Activation of membrane-associated receptors and intracellular signaling
Results	Reduced cytokine/chemokine production, decreased inflammatory cells	Rapid modulation of airway smooth muscle tone
Clinical relevance	Long-term control of inflammation	Rapid symptom relief

GR, glucocorticoid receptor.

The reduction in exacerbations observed with ICS-based rescue therapy is likely multifactorial. While suppression of airway inflammation could be a key contributor, additional mechanisms may also play important roles in reducing exacerbations, albeit these mechanisms have not been fully studied. One such mechanism may be the timely and repetitive use of as-needed ICS during periods of worsening symptoms, which may help to blunt the exacerbation (75). Another potential mechanism is the partial compensation for poor adherence to maintenance therapy with use of anti-inflammatory rescue at times of rising airway inflammation.

## 5 Conceptual basis for a shift in treatment for exacerbations

Since 2019, GINA has advocated for a move away from SABA-only rescue treatment for asthma (11). The rationale for supporting that all patients, even those with mild asthma, be provided with ICS-containing treatment from the start of therapy stems from GINA’s effort to provide consistent messaging about the need for both symptom relief and risk reduction and the need to reduce reliance on SABA therapy as the main asthma treatment, given its proven risks and limitations in all asthma populations (11, 63).

In addition to the aforementioned adverse outcomes associated with SABA-only therapy, the paradigm shift in rescue therapy was driven by the plethora of data from clinical trials demonstrating reductions in exacerbation risk with ICS-based rescue therapy (76–78). These and later studies provided the evidence base for the rescue therapy strategies introduced in the GINA expert report, including the use of SMART and AIR therapy across asthma severity (7).

### 5.1 SMART (MART) therapy

Randomized controlled trials (RCTs) have demonstrated that the use of budesonide–formoterol as both rescue therapy and as maintenance therapy in a SMART (MART) regimen was associated with reductions in severe exacerbations, ED visits, and hospitalizations, as well as reduction in exposure to SCS, compared with ICS–LABA maintenance plus as-needed SABA in patients with moderate-to-severe asthma (76–78). In one of these studies involving 2,760 patients, budesonide–formoterol used as a SMART regimen reduced the risk of severe exacerbations by 45–47% versus budesonide–formoterol maintenance therapy plus as-needed SABA (hazard ratio [HR], 0.55; 95% confidence interval [CI]: 0.44, 0.67) or budesonide maintenance therapy plus as-needed SABA (HR, 0.53; 95% CI: 0.43, 0.65) (76).

In another trial of 3,394 patients who were receiving budesonide–formoterol as maintenance therapy, as-needed budesonide–formoterol reduced the risk of a severe exacerbation by 27% versus formoterol (95% CI: 10%, 41%) and by 45% versus SABA (95% CI: 32%, 55%), underlining the beneficial role of ICS in patients who remain symptomatic despite treatment with combination maintenance therapy (78).

Furthermore, a meta-analysis of five RCTs of patients with poorly controlled asthma (*n* = 4,863) demonstrated that budesonide–formoterol used as maintenance and rescue therapy prolonged the time to first severe asthma exacerbation and reduced the risk of severe exacerbation in patients with uncontrolled asthma at GINA Step 3 (moderate asthma) or 4 (moderate-to-severe asthma) by 30% compared with ICS–LABA maintenance therapy plus as-needed SABA (HR, 0.70; 95% CI: 0.58, 0.85); this benefit was observed even when the ICS–LABA was used at a higher dose (79).

Moreover, the benefits of SMART (MART) with budesonide–formoterol versus standard therapy with budesonide–formoterol maintenance plus as-needed SABA did not appear to be associated with an increased risk of SABA overuse or long-term SCS burden, as one study demonstrated that the SCS exposure per year (mg prednisone equivalent) was 793.7 mg (893.1) with budesonide–formoterol maintenance and rescue therapy versus 772.1 mg (1,062.7) with standard therapy (*p* = 0.76) (80). Budesonide–formoterol forms the basis of the SMART (MART) regimen, with a single inhaler used both for daily maintenance to prevent chronic inflammation and as rescue therapy to relieve symptoms in patients with moderate-to-severe asthma (GINA Steps 3–5) (Table 1) (7). A key limitation of SMART (MART) is that it is not maintenance agnostic—since the same inhaler is used for both maintenance and rescue therapy, patients are restricted to ICS–formoterol as their maintenance therapy. Because of the increased risk of adverse events (AEs), ICS–formoterol used as rescue therapy should not be combined with a maintenance ICS–LABA regimen that contains a non-formoterol LABA (7, 81). In addition, as ICS–formoterol is not currently FDA-approved for use as maintenance and rescue or as rescue therapy in the US, PCPs or insurers who strictly follow label indications may be reluctant to use it as part of a SMART (MART) regimen or as rescue therapy alone (7).

### 5.2 AIR therapy

Following clinical trials supporting ICS–formoterol as part of the SMART regimen for moderate-to-severe asthma, subsequent trials evaluated ICS-based rescue therapy in patients with mild asthma (82, 83).

In the phase 3, 52-week SYGMA 1 trial of 3,836 patients with mild asthma, as-needed budesonide–formoterol reduced the annual rate of severe exacerbations by 64% versus as-needed SABA (rate ratio, 0.36; 95% CI: 0.27, 0.49) and by 17% versus budesonide maintenance therapy plus as-needed SABA (rate ratio, 0.83; 95% CI: 0.59, 1.16) (83). Overall, the ICS daily dose in the as-needed budesonide–formoterol group was 17% that of patients receiving budesonide maintenance plus as-needed SABA.

In the phase 3, 52-week SYGMA 2 trial of 4,215 patients with mild asthma, as-needed budesonide–formoterol was noninferior to budesonide maintenance therapy plus as-needed SABA in reducing the annualized rate of severe exacerbations (rate ratio, 0.97; upper one-sided 95% confidence limit, 1.16) (82). Importantly, the reduction in exacerbation risk was achieved with a substantially lower ICS daily dose with as-needed budesonide–formoterol (66 μg) versus budesonide maintenance therapy plus as-needed SABA (267 μg). Furthermore, the median number of days with SCS treatment was the same in each group.

In addition to evidence from RCTs on the use of as-needed budesonide–formoterol, open-label studies evaluating as-needed budesonide–formoterol provide further support of its efficacy and reflect real-world practice. In the 52-week open-label Novel START trial involving 668 adults with mild asthma, as-needed budesonide–formoterol reduced the risk of an exacerbation by 54% versus as-needed SABA (HR, 0.46; 95% CI: 0.29, 0.73) (84). Similarly, the 52-week PRACTICAL study in 890 adults with mild-to-moderate asthma showed that as-needed budesonide–formoterol reduced the risk of severe exacerbations by 31% (relative rate, 0.69; 95% CI: 0.48, 1.00; *p* = 0.049) versus maintenance ICS plus as-needed SABA (85).

Although most of the evidence for ICS-based rescue therapy comes from studies in adults, data demonstrating its efficacy in children and adolescents are also available. In the open-label ASIST trial comprising 206 African American children and adolescents aged 6–17 years with mild asthma, as-needed ICS taken with as-needed SABA was noninferior to physician-adjusted treatment (i.e., ICS maintenance therapy plus as-needed SABA) with regard to exacerbation risk and asthma control and was associated with lower ICS exposure (86). In addition, in the 52-week open-label CARE study of 360 children aged 5–15 years with mild asthma, as-needed budesonide–formoterol reduced the rate of moderate or severe asthma attacks by 52% versus as-needed SABA (relative rate, 0.48; 95% CI: 0.31, 0.74; *p* = 0.0010) (87).

These trials support the concept of AIR therapy, which involves administration of low-dose ICS with a fast-acting bronchodilator, ensuring that a patient receives symptom relief as well as a dose of an ICS to treat the underlying airway inflammation with every use (7).

#### 5.2.1 SABA–ICS fixed-dose combination rescue therapy

The next advancement in the evolution of AIR therapy was the development of the SABA–ICS fixed-dose combination rescue therapy with albuterol–budesonide in a single inhaler. US FDA approved in 2023, albuterol–budesonide is the first SABA–ICS combination treatment available for the as-needed treatment or prevention of bronchoconstriction and to reduce the risk of exacerbations in patients aged ≥18 years with asthma (15). The advantage of a SABA–ICS combination therapy is that it can be used as a rescue therapy with any maintenance therapy (16, 17).

The efficacy and safety of albuterol–budesonide was evaluated in randomized, phase 3 trials across asthma severity in the MANDALA, DENALI, and BATURA trials (16, 17, 88). Details of these studies and their key outcomes are summarized in Table 4 (15–17, 88).

**Table 4:** Pivotal clinical trials of albuterol–budesonide in patients with asthma (15–17, 88).

Study	Design and population	Intervention (FDA-approved dose)	Endpoint	Key outcomes in the FDA-approved patient population aged ≥18 years
MANDALA (15, 16)	Phase 3, randomized, 24–74 weeks duration; moderate-to-severe asthma; age ≥12 years	As-needed albuterol–budesonide 180/160 μg vs. albuterol alone 180 μg	Time to first severe asthma exacerbation	28% reduction in exacerbation risk with albuterol–budesonide 180/160 μg vs. albuterol 180 μg (HR 0.72; 95% CI: 0.6, 0.86; *p* < 0.001)
			Annualized severe exacerbation rate	24% reduction in annualized severe exacerbation rate with albuterol–budesonide 180/160 μg vs. albuterol 180 μg (0.46 vs. 0.60, respectively; RR 0.76; 95% CI: 0.62, 0.93; *p* = 0.008)
			Annualized SCS dose	32% reduction in annualized SCS dose with albuterol–budesonide 180/160 μg vs. albuterol 180 μg (86.6 mg vs. 127.1 mg, respectively; *p* = 0.001)
BATURA (15, 17)	Phase 3b, decentralized, event-driven, 12–52 weeks duration; mild asthma; age ≥12 years	As-needed albuterol–budesonide 180/160 μg vs. albuterol alone 180 μg	Time to first severe asthma exacerbation	46% reduction in exacerbation risk with albuterol–budesonide 180/160 μg vs. albuterol 180 μg (HR 0.54; 95% CI: 0.41, 0.72; *p* < 0.001)
			Annualized severe exacerbation rate	54% reduction in annualized severe exacerbation rate with albuterol–budesonide 180/160 μg vs. albuterol 180 μg (0.15 vs. 0.33, respectively; RR 0.46; 95% CI: 0.33, 0.63; *p* < 0.001)
			Annualized SCS dose	63% reduction in annualized SCS dose with albuterol–budesonide 180/160 μg vs. albuterol 180 μg (23 mg vs. 63 mg, respectively)
DENALI (15, 88)	Phase 3, randomized, 12 weeks; mild-to-moderate asthma; age ≥12 years	Albuterol–budesonide 180/160 or 180/80 μg vs. albuterol 180 μg, budesonide 160 μg, or placebo	Change from baseline in FEV₁ AUC₀–₆_h_ over 12 weeks	LSM change: 251.8 mL with albuterol–budesonide 180/160 μg versus 174.6 mL with budesonide 160 μg; difference in LSM (95% CI), 77.2 (24.8, 129.6)
			Change from baseline in trough FEV_1_ at Week 12	LSM change: 127.1 mL with albuterol–budesonide 180/160 μg versus −2.0 mL with albuterol 180 μg
			Time to onset of bronchodilation, median	7.5 min with albuterol–budesonide 180/160 μg versus 10.0 min with albuterol 180 μg Onset of bronchodilation^a^: observed in 51% of patients in the albuterol–budesonide 180/160 μg group versus 43% of patients in the albuterol 180 μg group
			Duration of bronchodilation, mean	186.9 min with albuterol–budesonide 180/160 μg versus 167.9 min with albuterol 180 μg

AUC_0–6h_, area under the curve from 0 to 6 h; CI, confidence interval; FDA, US Food and Drug Administration; FEV_1_, forced expiratory volume in 1 s; HR, hazard ratio; RR, rate ratio; SCS, systemic corticosteroid. ^a^As defined by a ≥ 15% increase in FEV_1_ post-dose within 30 min on Day 1.

In the MANDALA trial of 3,132 patients with moderate-to-severe asthma, albuterol–budesonide 180/160 μg versus albuterol 180 μg reduced the risk of a severe asthma exacerbation by 28% (*p* < 0.001), the annual rate of severe exacerbations by 24% (*p* = 0.008), and the mean annualized SCS dose by 32% (86.6 mg vs. 127.1 mg, respectively; *p* = 0.001) among the FDA-approved patient population aged ≥18 years (15, 16). The exacerbation reduction was observed among patients with high adherence to maintenance therapy (16), confirming that the extra ICS administered as rescue therapy produced an added benefit on top of ICS-containing maintenance therapy. The increased use of as-needed medications around the time of clinical deterioration of asthma shown in MANDALA, with a subsequent reduction in exacerbations, provides proof of concept for the hypothesis that delivery of ICS during the window of opportunity prior to an exacerbation decreases the likelihood of a full-blown exacerbation (16). The incidence of AEs was similar across treatment groups, with the most common AEs being nasopharyngitis, headache, and upper respiratory tract infections (16).

In the BATURA trial of 2,516 patients with mild asthma, patients who received albuterol–budesonide 180/160 μg versus albuterol 180 μg among the FDA-approved patient population aged ≥18 years experienced a 46% reduction in the risk of a first severe exacerbation (95% CI: 0.41, 0.72), a 54% reduction in the annualized rate of severe asthma exacerbations (95% CI: 0.33, 0.63), and a 63% reduction in SCS dose (23 mg vs. 63 mg, respectively). The majority (88.8%) of patients had no severe exacerbations in the 12 months prior to trial entry, confirming that the benefits of albuterol–budesonide rescue therapy are not confined to patients at high risk of exacerbations (17). As in the MANDALA trial, the frequency of AEs was similar across treatment groups, and the most common AEs included respiratory tract infection, COVID-19, and nasopharyngitis (17).

## 6 Expert opinion report for rescue therapy

The most current GINA strategy report (7) and the National Asthma Education and Prevention Program (NAEPP) guidelines (18) differ with regard to their guidance for ICS-based rescue therapy (Table 5) (89).

**Table 5:** Strategies for incorporating ICS into rescue therapy (7, 18).

NAEPP focused updates 2020 (18)	GINA 2025 (7)
Step 2 (patients aged ≥12 years): daily low-dose ICS and as-needed SABA or as-needed concomitant ICS and SABA	Track 1 (patients aged ≥12 years): as-needed low-dose ICS–formoterol* as rescue therapy at Steps 1–5
Steps 3 and 4 (patients aged ≥4 years): daily and as-needed combination ICS–formoterol	Track 2 (patients aged ≥12 years): as-needed SABA+ICS at Step 1 (in a combination inhaler or in separate inhalers) and SABA–ICS* combination therapy at Steps 2–5

*As-needed SABA rescue therapy to be used only if the ICS–formoterol or SABA–ICS combination therapy is unavailable.

GINA, Global Initiative for Asthma; ICS, inhaled corticosteroid; NAEPP, National Asthma Education and Prevention Program; SABA, short-acting beta_2_-agonist.

NAEPP recommends as-needed SABA monotherapy at Step 1 (intermittent asthma) across all ages (18). At Step 2 (mild persistent asthma), daily low-dose ICS and as-needed SABA can be used across all ages, or as-needed concomitant ICS and SABA for patients aged ≥12 years. SMART therapy with low-dose or medium-dose ICS–formoterol is the preferred treatment option at Step 3 (moderate persistent asthma) or Step 4 (moderate-to-severe persistent asthma), respectively, in patients aged ≥4 years (18). At Step 5 (severe persistent asthma), the preferred treatment option for patients aged <12 years is daily high-dose ICS-based therapy and as-needed SABA, while daily medium- or high-dose ICS–LABA plus long-acting muscarinic antagonist (LAMA) and as-needed SABA are recommended for patients aged ≥12 years. Across all ages, daily high-dose ICS–LABA plus oral SCS and as-needed SABA is recommended at Step 6. It is important to note that the NAEPP guidelines, last updated in 2020, are based on reviewed data published only up to 2018 (18), and that the evidence base for SABA–ICS therapy with albuterol–budesonide across all disease severities was published after the 2020 NAEPP guidelines were released.

The GINA 2026 report supports as-needed low-dose ICS–formoterol as the preferred rescue therapy (Track 1) for symptom relief for patients aged ≥12 years across the disease severity spectrum (7). AIR-only treatment with low-dose ICS–formoterol should be used at GINA Steps 1 and 2, followed by SMART (MART) with ICS–formoterol at GINA Steps 3–5. But as previously mentioned, ICS–formoterol is not approved for use as rescue therapy alone or as SMART (MART) in the US or with maintenance therapies utilizing a LABA other than formoterol (7, 81). Therefore, for patients unable to take low-dose ICS–formoterol for any reason, GINA supports Track 2, an alternative approach in which rescue includes an ICS used concomitantly with SABA across all steps of therapy (7): At Step 1, patients taking SABA as rescue therapy should concomitantly take low-dose ICS for symptom relief, administered in either a combination inhaler or in separate inhalers; no maintenance ICS is needed at Step 1. However, daily maintenance ICS in some form should be added to as-needed SABA–ICS combination therapy at Steps 2–5. It is important to note that GINA supports use of as-needed SABA only if as-needed SABA–ICS combination therapy is unavailable; clinicians should carefully assess a patient’s likelihood of adhering to maintenance therapy before prescribing SABA rescue therapy, as poor adherence to maintenance treatment increases the risk of exacerbations (7).

Asthma management in children differs from that of adolescents and adults due to diagnostic uncertainty, safety considerations, reliance on caregiver-supported inhaler use, and a more limited evidence base for certain treatment strategies (7). As in adolescent and adult patients, GINA advises against use of SABA-only therapy in children aged 6–11 years, even at GINA Step 1; rather, AIR therapy with low-dose ICS–formoterol or low-dose ICS with SABA (taken either in a combination inhaler or in separate inhalers) is the preferred GINA Step 1 treatment; daily low-dose ICS plus as-needed SABA–ICS combination therapy at GINA Step 2; and daily maintenance ICS in some form plus as-needed SABA–ICS, or low-dose SMART (MART) with ICS–formoterol at GINA Steps 3–5. Similar to the guidance for adolescents and adults, GINA supports as-needed SABA rescue therapy only if as-needed SABA–ICS combination therapy is unavailable (7).

Safety regarding ICS use is an important consideration. Local AEs following ICS use may include dysphonia and oropharyngeal candidiasis, while long-term high doses of ICS may increase risk of systemic AEs, such as glaucoma, cataract, and osteoporosis (7). The safety of ICS is an especially important factor in children, as reduction in growth velocity has been reported in children taking ICS in the first 1–2 years of treatment. As such, because the effect of ICS on growth is dose dependent, GINA supports use of the minimum effective dose to maintain asthma control (7). However, the benefits of ICS-based rescue therapy must be balanced against potential risks: ICS-based rescue therapy significantly reduces the risk of exacerbations, as well as the likelihood of SCS exposure—a key benefit, given the myriad of systemic AEs associated with SCS use, as noted previously (7, 49).

Despite differences in guidance between GINA and NAEPP, all treatment strategies recognize that deaths and exacerbations can occur in patients across the range of asthma severities. Both the GINA reports and the NAEPP guidelines concur that ICS therapy is foundational for asthma treatment and that there is benefit to ICS rescue therapy (7, 18).

## 7 Importance of SABA–ICS therapy in clinical practice

Addressing exacerbations is critical, given that they lead to increased morbidity (90) and increased SCS use and associated adverse effects (46, 48, 49). Broad use of a SABA–ICS combination inhaler as rescue therapy across all disease severities for patients ≥18 years has the potential to decrease the rate of exacerbations and reduce SCS use (16, 17). Unlike SMART, SABA–ICS combination therapy with albuterol–budesonide is (1) FDA-approved for as-needed treatment in patients aged ≥18 years with asthma (15); and (2) maintenance therapy agnostic; therefore, it can be used in place of albuterol with all FDA-approved maintenance therapies.

Although real-world studies have yet to be conducted, it is possible that the reduction in exacerbations with albuterol–budesonide use in real-world practice will be greater than that seen in RCTs. This speculation is largely driven by recognizing the gap in adherence rates to ICS-based maintenance therapy in RCTs versus those in real-world studies. RCT participants are generally more adherent to treatment than most patients in clinical practice, as was evident in the MANDALA trial, in which the mean adherence to maintenance therapy exceeded 75% (91). In the real world, pharmacovigilance data show that patients with asthma typically use only 40–50% of prescribed ICS maintenance doses (i.e., approximately 4–6 months of the prescribed 12 months), with approximately 4.7–5.5 fills/year (92). A small proportion of patients (~8%) may never fill their ICS prescriptions (93). Furthermore, a systematic literature review of 19 observational and interventional studies found that adherence to ICS-based maintenance therapy ranged from 22% to 63% (39), with patients preferring to use less maintenance therapy when asthma is controlled and increasing use when asthma worsens (68, 94). Thus, because significant reduction in exacerbation risk was reported with albuterol–budesonide in patients using maintenance therapies appropriate for moderate-to-severe asthma who were adherent to these treatments (16), it is possible to infer that delivery of as-needed ICS will have a greater effect among the group of patients in the real world who do not have adequate utilization of anti-inflammatory maintenance therapy. In addition, the use of a fixed-dose combination inhaler may help to overcome the issues associated with poor ICS adherence, ensuring that ICS therapy is given when it is most needed to treat underlying inflammation and prevent exacerbations (89).

Potential future research areas include investigation of SABA–ICS in pediatric and adolescent patients. Studies assessing the real-world effectiveness and safety of SABA–ICS will also be useful.

## 8 Conclusion

New evidence regarding the efficacy of albuterol–budesonide in reducing exacerbation rates across all asthma severities has created an opportunity in the US to align rescue therapy strategies with the long-standing approach of using an anti-inflammatory medication for maintenance therapy to mitigate the underlying variable airway inflammation that characterizes asthma and results in symptoms and exacerbations. Timely delivery of an ICS—when its anti-inflammatory effects may ameliorate the ongoing inflammatory cascade—has been demonstrated to reduce the likelihood of initial symptoms turning into a full-blown exacerbation, reducing both exacerbation occurrence and SCS exposure.
